# Nonsuicidal self-injury: a condition for further study

**DOI:** 10.1186/s13034-015-0067-2

**Published:** 2015-08-11

**Authors:** Paul L Plener, Joerg M Fegert

**Affiliations:** Department of Child and Adolescent Psychiatry and Psychotherapy, University Hospital, Steinhövelstr.5, 89075 Ulm, Germany

Although nonsuicidal self-injury (NSSI) has been recognized in the scientific literature since the 1960s [1, 2] with ever increasing rates of publications since, there has been some dispute about the correct classification, and definition [3–5], with different terms, making it difficult to compare data between studies. The Diagnostic and Statistical Manual (DSM-5) of the American Psychiatric Association, published in 2013 marked a next step in this debate, as it included a section named “conditions for further study”, presenting criteria for condition “on which future research is encouraged”, which were “not intended for clinical use” [6]. The aim of developing NSSI as a separate syndrome and setting it apart from suicidal behaviors was—and still is—not undisputed [7–11]. The criteria that were used in the “conditions for further study” section, were “set by expert consensus—informed by literature review, data reanalysis, and field trial results” and were “intended to provide a common language for researchers and clinicians who are interested in studying these disorders” [6]. The DSM-5 “condition for further study” approach was chosen to spark new research “to better understand these conditions” and “inform decisions about possible placement in forthcoming editions of DSM” [6].

This is exactly what this thematic series adds: a wide range of new research increasing the knowledge we currently have about NSSI, going beyond the borders of the already known. It is a sign of an emerging scientific field that still, many questions are unanswered, thus allowing and longing for a lively and proactive scientific community. To fill the gaps in knowledge, a group of researchers has devoted itself to gather new insights in NSSI and establish a sound knowledge base. The members of the International Society for the Study of Self Injury (ISSS) (http://www.itriples.org), have—since the society´s first meeting in 2006—been actively involved in research on NSSI around the globe. The 10th annual meeting of the ISSS, which was held in Heidelberg in June 2015, was inducement to create this special issue. However, this is not an issue looking back at goals, which already have been achieved and knowledge that was built through past research, but it is an issue that further adds to this body of knowledge, by presenting new data and systematic reviews of existing research. The sheer quality and quantity of manuscripts handed in after our call, allowed us to create a two-volume issue on NSSI, unprecedented in the history of Child and Adolescent Psychiatry and Mental Health. Taking a closer look at the studies, which appear in the first volume of this thematic series, we can safely claim, that a broad variety of topics is covered:

Questioning what maintains and what stops these behaviors, Duggan et al. present data from a one-year longitudinal study. The authors reported from a sample of young adolescents (between the age of 11 and 13), that in comparison to participants stopping NSSI, those maintaining NSSI, showed greater body surveillance, as well as more emotional dysregulation and depressive symptoms, findings which are discussed with relation to objectification theory. Also focusing on cessation of NSSI, the study of Whitlock et al. (2015) presents data on students ending NSSI, suggesting that changes in emotion regulation strategies, as well as increasing self-awareness, maturity and good connections with others are crucial points in the process of cessation of NSSI, a process, which can be aided by therapy. The cessation of NSSI is often strongly linked to the question of help-seeking behavior for NSSI, which was studied by Pumpa and Martin. The authors were able to show, that a more positive attitude towards help seeking differentiated current from past self-injurers or participants without a history of NSSI. Interestingly, participants with NSSI, who sought help were more likely to approach informal than formal sources—a finding, which should be taken into consideration when tailoring future interventions to the needs of people with NSSI.

Adding further knowledge about factors influencing NSSI, Garisch and Wilson report data from a large longitudinal study of a community sample of adolescents in New Zealand, replicating findings from smaller cross-sectional studies in the past and reporting an association of NSSI with (among others) bullying, anxiety, depression, alexithymia, a history of sexual abuse, lower self-esteem and concerns about sexuality. Further inquiring the question of sexual minority status in relationship to NSSI, Muehlenkamp et al., present data of 137 college students identifying themselves as a member of a sexual minority group. The study showed, that stressors resulting from a sexual minority status (“minority stress”) are directly associated with NSSI.

While research shows an increasing number of large community based studies, data on large clinical samples are still rare in NSSI research, so that a data set of 1520 adolescents and young adult patients treated in a self-injury program as presented in the paper by Victor et al., allows for insights, that are unprecedented so far in NSSI literature. Studying the relationship between suicidal ideation and NSSI, Victor and colleagues described a strong association between suicidal ideation and low severity methods of NSSI, as well as a strong association between suicidal ideation and intrapersonal functions of NSSI. Adding more data from an inpatient sample of adolescents, Nixon et al. described the Ottawa Self-Injury Inventory (OSI) as a valid and reliable assessment tool able to provide information on the four factor model of NSSI, as well as on addictive features.

A growing body of recent literature has put an emphasis of familial relationships of adolescents injuring themselves. Following this line of research, Tschan et al. present data about parenting behaviors in families of female adolescents injuring themselves and compares them with families of adolescents from both a clinical and healthy control group. In families of adolescents with NSSI, less maternal warmth and support was reported, than in healthy controls. Also with regards to families and NSSI, the work of Baetens et al., complements this picture by offering insights into the parental perspective in the first prospective study on this issue ever published. The authors were able to show a reciprocal relationship between parenting behavior and NSSI in a longitudinal approach, with a reciprocal effect of e.g. controlling parenting behavior and NSSI over time.

Following our first NSSI thematic series in 2012, which anticipated the upcoming NSSI definition in DSM-5 [12], we hope that this two volume special issue will find as many readers (or even more) as our first approach to cover new research in this field with a broad spectrum of new studies.
